# Health outcomes, education, healthcare delivery and quality – 3047. What do patients think about asthma-specific quality of life questionnaires? Qualitative interview study in the UK

**DOI:** 10.1186/1939-4551-6-S1-P217

**Published:** 2013-04-23

**Authors:** CJ Apfelbacher, Anthony Frew, Helen Smith

**Affiliations:** 1 Brighton & Sussex Medical School, UK; 2 Brighton & Sussex University Hospitals NHS Trust, Brighton, UK

## Background

The purpose of this study was to explore patients’ views on the content of three asthma-specific quality of life questionnaires.

## Methods

22 adult individuals with asthma were asked to complete three asthma-specific quality of life questionnaires (Juniper Asthma Quality of Life Questionnaire (AQLQ-J), Sydney Asthma Quality of Life Questionnaire (AQLQ-S), Living With Asthma Questionnaire (LWAQ)). Interviews were conducted to elicit patients’ views on the content validity of the questionnaires and transcribed verbatim. Thematic content analysis was performed.

## Results

Participants spoke about missing content, redundant or similar content, irrelevant content, confusing content and irritating content. The AQLQ-J was perceived as a rather ‘narrow’ and ‘medical’ questionnaire with a focus on the environment and activities. The choice of activities was perceived to be positive by some and difficult by other participants. The AQLQ-J was contrasted with both the LWAQ and the AQLQ-S which were perceived to be ‘non-medical’, wide-ranging questionnaires with a social and emotional focus. The emotional focus of the LWAQ as well as the use of positive and negative items were perceived as irritating by some participants and overall, it was perceived as burdensome to complete and described as a ‘test’ or ‘quiz’. In contrast, the AQLQ-S was described as a simple, quick and easy questionnaire although it was thought to lack depth at the same time. Overall, the AQLQ-S was situated ‘between’ the AQLQ-J as a ‘medical’ questionnaire and the LWAQ as an ‘emotional’ questionnaire.

## Conclusions

Patient involvement highlights shortcomings and strengths of various asthma-specific questionnaires in terms of their content validity. The AQLQ-S seems to have acceptable length and yet sufficient coverage of medical, social and emotional aspect of health-related quality of life in asthma.

